# Myogenic and cortical evoked potentials vary as a function of stimulus pulse geometry delivered in the subthalamic nucleus of Parkinson’s disease patients

**DOI:** 10.3389/fneur.2023.1216916

**Published:** 2023-08-24

**Authors:** Brett A. Campbell, Leonardo Favi Bocca, Jakov Tiefenbach, Olivia Hogue, Sean J. Nagel, Richard Rammo, David Escobar Sanabria, Andre G. Machado, Kenneth B. Baker

**Affiliations:** ^1^Department of Biomedical Engineering, Case Western Reserve University, Cleveland, OH, United States; ^2^Department of Neurosciences, Cleveland Clinic, Cleveland, OH, United States; ^3^Center for Neurological Restoration, Cleveland Clinic, Cleveland, OH, United States; ^4^Center for Quantitative Health Sciences, Cleveland Clinic, Cleveland, OH, United States; ^5^Department of Neurosurgery, Cleveland Clinic, Cleveland, OH, United States; ^6^Department of Biomedical Engineering, Cleveland Clinic, Cleveland, OH, United States

**Keywords:** deep brain stimulation, Parkinson’s disease, cortical evoked potentials, pulse geometry, motor evoked potential, subthalamic nucleus

## Abstract

**Introduction:**

The therapeutic efficacy of deep brain stimulation (DBS) of the subthalamic nucleus (STN) for Parkinson’s disease (PD) may be limited for some patients by the presence of stimulation-related side effects. Such effects are most often attributed to electrical current spread beyond the target region. Prior computational modeling studies have suggested that changing the degree of asymmetry of the individual phases of the biphasic, stimulus pulse may allow for more selective activation of neural elements in the target region. To the extent that different neural elements contribute to the therapeutic vs. side-effect inducing effects of DBS, such improved selectivity may provide a new parameter for optimizing DBS to increase the therapeutic window.

**Methods:**

We investigated the effect of six different pulse geometries on cortical and myogenic evoked potentials in eight patients with PD whose leads were temporarily externalized following STN DBS implant surgery. DBS-cortical evoked potentials were quantified using peak to peak measurements and wavelets and myogenic potentials were quantified using RMS.

**Results:**

We found that the slope of the recruitment curves differed significantly as a function of pulse geometry for both the cortical- and myogenic responses. Notably, this effect was observed most frequently when stimulation was delivered using a monopolar, as opposed to a bipolar, configuration.

**Discussion:**

Manipulating pulse geometry results in differential physiological effects at both the cortical and neuromuscular level. Exploiting these differences may help to expand DBS’ therapeutic window and support the potential for incorporating pulse geometry as an additional parameter for optimizing therapeutic benefit.

## Introduction

Deep brain stimulation (DBS) of the subthalamic nucleus (STN) is standard of care treatment for motor symptoms of moderate to severe Parkinson’s disease (PD) (1, 2). To optimize therapeutic benefit and minimize side effects, a clinician is able to refine settings related to the location, frequency, amplitude, and width of the electrical pulses delivered via the DBS lead. The shape, or geometry, of the individual biphasic pulses themselves is fixed, however, with a charge-balanced, cathode-leading asymmetric pulse standard on currently-approved devices (3, 4). This limitation persists despite prior computational modeling work suggesting that manipulating pulse geometry may yield differences in the neural elements affected by stimulation and serve to enhance therapeutic selectivity (5, 6). While there is recent evidence to suggest that flipping the polarity of the current DBS pulse can influence therapeutic window (7), no study has investigated whether manipulating pulse geometry alters physiological responses that can be recorded from PD patients with STN-DBS implants. Understanding how pulse shape impacts both the cortical and muscular response patterns associated with STN-DBS may help us to understand how geometry influences selectivity *in vivo* and facilitate the development of neural element-specific techniques to optimize therapeutic outcomes.

Previous modeling work by McIntyre and Grill proposed manipulating pulse geometry as a means of enhancing neural selectivity during electrical stimulation (6). The authors introduced a long-duration and short amplitude conditioning pre-pulse phase prior to a short-duration and large amplitude primary stimulation phase to influence the threshold for small and large-diameter fibers through changes in the current distance relationship (6, 8). Their work suggests that manipulating the degree of asymmetry of the pulse phases combined with reversing polarity can result in enhanced cell body vs. axonal recruitment that is absent when stimulating with symmetric biphasic pulses. *In-vivo* preclinical studies have further explored and supported this phenomena and demonstrated enhanced spatial selectivity as a result of manipulating pulse geometry (9). No clinical studies have yet explored how such changes in pulse geometry may impact the physiological response to stimulation in STN-DBS for PD.

The physiological effects of DBS in the region of the STN can be appreciated using standard electromyography (EMG) (10–14) and scalp electroencephalography (EEG) (15, 16) based techniques. Muscle contraction represents one of the most common, amplitude-limiting side effects associated with STN DBS. It typically affects muscles of the lower face or distal upper extremity and is attributable to spread of electrical current laterally to the corticofugal fibers that make up the adjacent internal capsule. Clinically, such effects are readily appreciated or reported as twitches or tightening of the affected body region; however they can be further characterized quantitatively using stimulus-locked EMG to record myogenic evoked potentials (MEPs) that generally occur approximately 20 ms after stimulus delivery (10). The precise origin of the EEG-based, DBS cortical evoked potential (DBS-CEPs) is less clear as the response likely represents a composite of various neural elements, and corresponding pathways, activated in the region of the DBS lead (15–17). Since its introduction, a number of studies have characterized how the DBS-CEP activation pattern changes in relation to specific DBS parameters (18–20), patient status (21), and as a function of overall therapeutic benefit (22–24). The short latency responses (i.e., those occurring within a few milliseconds from the onset of stimulation) recorded over motor cortex have, for example, been hypothesized to reflect antidromic activation of the hyperdirect pathway between motor cortical regions and STN (20, 24, 25) and further posited to represent the primary therapeutic pathway of STN-DBS (20, 24, 26–28). Manipulating the pulse geometry of the electrical stimulus waveform may allow for enhanced targeting of potential therapeutic pathways and the avoidance of those associated with side effects.

In this study, we investigated how manipulating the degree of asymmetry of the pulses phases (i.e., pulse geometry) of STN DBS influenced myogenic and scalp-recorded DBS-CEPs (short and long-latency) in patients with PD. For the MEP response, we proposed that the pulse geometries identified by McIntyre and Grill as preferentially activating axons, would show earlier (i.e., at lower pulse amplitudes) recruitment of corticofugal fibers as reflected by the DBS-MEP in comparison to those predicted to be selective for cell bodies (6). For the DBS-CEP, our work was guided by a recent pair of computational modeling studies suggesting that specific pulse geometries should alter the recruitment of the terminating fibers of the hyperdirect pathway during STN-DBS (29, 30), which would be reflected in the appearance of short-latency CEPs. We explore how the long-duration and small amplitude anodic phase followed by a short-duration and large amplitude cathodic phase proposed may be selective for axons and capable of producing larger MEP responses during monopolar stimulation than their cell body recruiting counterpart. We also investigate how short-latency cortical responses reflecting antidromic activation of the hyperdirect pathway may show preferential activation based on changes in pulse geometry. Our data provide insights into how we can minimize DBS-induced motor side effects and potentially increase the therapeutic window through manipulating DBS pulse geometry.

## Materials and methods

### Data acquisition and analysis

#### Participants

The Cleveland Clinic Institutional Review Board (IRB) approved all research and participants provided written informed consent prior to participating (NCT04563143) in accordance with the Declaration of Helsinki. Data reported in this study are derived from patients who underwent standard of care unilateral (participant 6) or staged, bilateral (all other participants) DBS lead implantation surgery targeting the STN for the treatment of PD. For each participant, a single DBS lead was externalized for up to 9 days prior to implantable pulse generator (IPG) placement to allow for data collection. Data from the current study were collected between days three through eight post-surgery with patient seated in a recliner and in the dopaminergic medication ON-state.

#### Anatomical localization of leads

**Figure 1:**
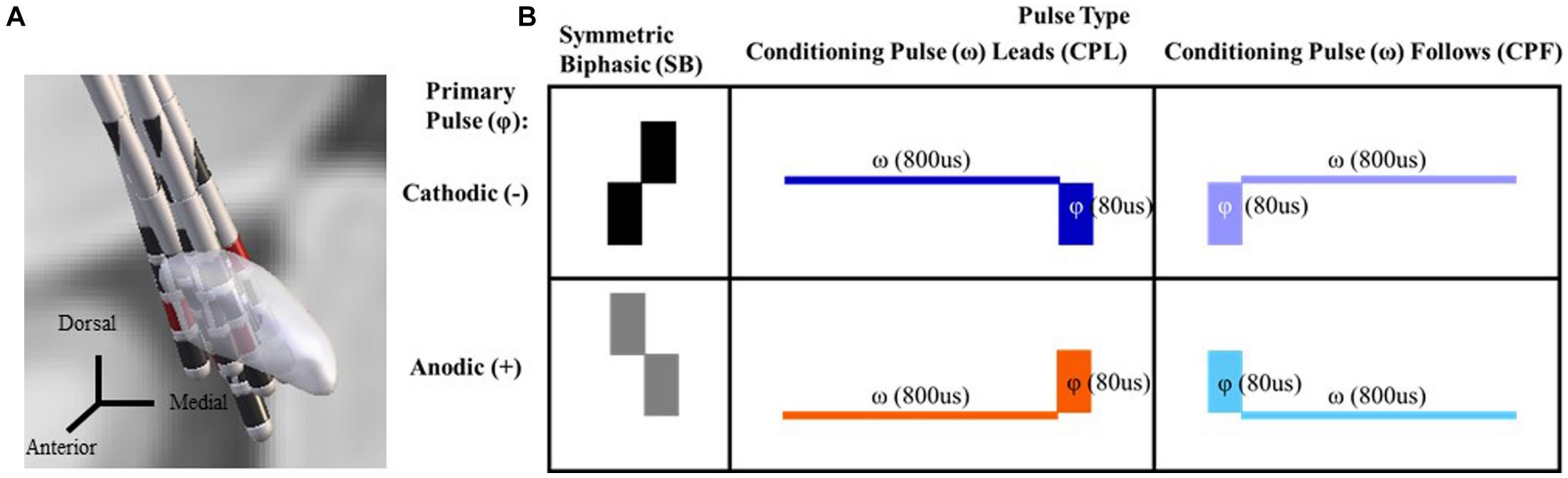
DBS lead localization for each participant via Lead DBS. Coronal view looking posteriorly with the cathode selected through monopolar review shown in red **(A)**. A chart reflecting the six pulse geometries utilized in this study. The symmetric biphasic geometries are shown in the furthest two left panels with the cathode leading version (SB−) above the anode leading version (SB+). In the asymmetric pulse types the primary phase is denoted by a phi symbol. A conditioning phase is denoted by a omega symbol and either leads or follows the primary phase (referred to as the CPL+ and CPF− conditions, respectively). The geometry is cathodic or anodic based on the orientation of the primary phase (i.e., CPF− for the top right panel and CPF+ for the bottom right; **B**).

DBS lead localization was performed post-operatively using the MATLAB toolbox Lead DBS v2.5.3, in line with the methodology previously described in Horn et al. (31). The pre-operative volumetric T1-weighted with contrast MRI and post-operative head CT were used. The images were uploaded in DICOM format and converted to NIfTI file with the dcm2niix protocol (32). The post-operative head CT was co-registered to pre-operative MRI using a two-stage linear registration (i.e., rigid followed by affine registration) as implemented in Advanced Normalization Tools (33). Subsequently, the preoperative acquisitions were spatially normalized into MNI ICBM 2009b nonlinear asymmetric space (34) using a three-step non-linear affine registration (33, 35). Localization of the lead was done in the co-registered postoperative head CT, manually matching the lead’s model to the acquired image. Contact coordinates were calculated at their geometric center in MNI space coordinates. Using MNI to ACPC algorithm implemented by Horn et al., MNI coordinates were converted to coordinates relative to midcommisural point (36). Coordinates x, y, and z represent the lateral, anterior, and inferior distance, respectively, between the contact and the MCP, expressed in millimeters and rounded to the nearest one decimal place. For representation purposes, left-side leads were moved to the right side flipping coordinate X from the left to right side. The “DISTAL” brain atlas was used for STN segmentation in MNI space (37). The resulting co-registration for each participant’s DBS lead relative to the STN target is shown in Figure 1A.

#### Contact selection

All participants underwent a monopolar review of the externalized lead between 1 and 3 days post-operatively. This process identifies the stimulation contacts on the DBS lead best associated with clinical improvement as not all contacts on an implanted lead will provide therapeutic benefit. We chose to focus our experimental efforts on the contact (s) selected through monopolar review. A constant rate of 130 Hz and pulse width between 60 and 90 μs were used. For each contact, the amplitude of stimulation was gradually increased in pre-defined increments of 0.5 (Volts for Medtronic system and milliamperes for others), and the threshold, localization, subjective intensity (i.e., low, medium, high), and duration (i.e., transient or continuous) of the side-effect(s) were documented. The maximum amplitude of stimulation before non-transient side-effects (low intensity) was defined as the stimulation threshold. Efficacy assessments were performed similarly for each contact by a trained clinician, guided primarily by improvements in rigidity and tremor while the participant was OFF-medication. The active contact for stimulation used to characterize the electrophysiological data was determined based on the widest therapeutic window. For the bipolar configuration portion of the study the adjacent contact (or contacts when a pseudo-annular ring was required) along the Z dimension of the lead with the next most effective therapeutic window was selected as the return.

#### Pulse geometry and stimulation parameters

A total of six pulse geometries were used in this study (Figure 1B) and delivered in both a monopolar and bipolar montage across the selected contacts from the monopolar review findings as described above. Delivering both in a bipolar and monopolar configuration allowed for evaluation of whether the differences in the electric field influenced the effects of manipulating pulse geometry. The first two pulse geometries were symmetric biphasic (SB) and differed in terms of the order in which the cathodic and anodic phases were delivered (SB− and SB+, respectively). This configuration is commonly used in preclinical and research contexts, as it allows for the use of phase reversal to help differentiate the end of the stimulus artifact from the start of the physiological response in DBS-CEPs (16). The lowest amplitude was 0.6 mA and the upper limit did not exceed 7.0 mA. Pulse width was set constant for each phase of the SB condition at 80us. Two sets of asymmetric configurations were evaluated. This included a conditioning phase leads (CPL) asymmetric pulse geometry that was based on dimensions from McIntyre and Grill (6) where the first phase served as a conditioning pre-pulse (ω) and was 10x the width (i.e., 800us) and 1/10 of the amplitude (i.e., 0.06–0.70 mA) of the primary phase (φ) (6). The phase orientation was also evaluated in a reversed configuration, without a conditioning pre-pulse, such that the lower amplitude wider phase pulse followed (CPF) the primary phase and served only as a charge balancing phase. This configuration approximates the pulse geometry utilized in standard of care, clinical implantable pulse generators. Each orientation of the asymmetric pulse geometry was delivered such that the primary phase was tested in both anodic and cathodic form (Figure 1B: referred to as CPL+ and CPL−; CPF+ and CPF−, respectively). It should be noted, however, that the effects of polarity reversal for adjacent contacts in a bipolar stimulation montage negate the effects of phase order rendering them no longer purely anodic vs. cathodic. The reference to whether stimulation is anodic vs. cathodic for the bipolar montage going forward refers exclusively to the polarity of the primary phase (φ) observed across the contact(s) selected during monopolar review. Stimulation was delivered using the Subject Interface Stimulator from Tucker Davis Technologies (Alachua, FL, United States). For the monopolar configuration, an electrode patch was adhered to the participant’s chest above the eventual position of the IPG and served as the electrical return. Stimulation was performed at 5.1 Hz to avoid interactions with 60 Hz line noise and corresponding harmonics as well as to maximize the amount of samples acquired within a fixed period of time.

#### Data acquisition

Myogenic evoked potentials (MEPs) were used to evaluate the effect pulse geometry on activation of the adjacent internal capsule fibers (10–13). Electromyography electrodes from Medsource Labs (Chanhassen, MN, United States) were placed on the arm contralateral to stimulation with differential recordings across the deltoids, biceps, triceps, flexor carpi radialis (FCR), and extensor digitorum communis (EDC) muscles and a ground electrode placed on the sternum. Scalp electroencephalography (EEG) was acquired to understand the effect of manipulating pulse geometry on the short and long-latency DBS-CEP components given their putative relationship to therapeutic outcomes and mechanisms of STN-DBS (11, 20, 23, 26–28, 38). Silver/silver chloride surface electrodes were placed according to the standard 10–20 EEG montage, with additional electrodes at positions FC1, FC2, FC5, FC6, FT9, CP1, CP2, CP5, CP6, TP9, and TP10. EEG data were left–right flipped to align to a common (right) side. All data were acquired using a Tucker Davis Technologies 128-channel electrophysiology recording system (Alachua, FL, United States) and sampled at ~24KHz with an antialiasing filter whose cutoff frequency was equal to 45% of the sampling rate.

#### Signal processing

Prior to averaging, periods of muscle or other artifact contamination were removed automatically via custom scripts in MATLAB. The full trace from the C4 electrode (referenced only to Pz for this step) was band pass filtered in the 8–100 Hz range and chosen for artifact detection based on its proximity to the brain regions of interest discussed below. We observed infrequent activity by participants (i.e., coughs, shifting their weight in the chair, etc.) that would create 1–2 s artifacts during data collection that were excluded from analysis. In anticipation of this we collected up to 750 stimulation pulses, but were able to generate consistent evoked responses with fewer pulses. Thus, the minimum amount of summed pulses after artifact rejection across a few amplitudes and dispersed throughout each condition (CPL−/+, CPF−/+, SB−/SB+ in both the monopolar and bipolar configurations) was 450 pulses. The majority of averages exceeded 500 stimulation pulses. In circumstances where data acquisition personnel noted sustained artifacts during collection a trial was repeated to ensure at least 450 clean stimulation pulses. Instances where the amplitude of the signal exceeded 20uV were marked as periods of artifact contamination that were excluded from the evoked response calculation (see Supplementary Figure 1). Visual inspection of individual trials was also performed to validate the automated artifact removal. The composite average evoked potential for each pulse geometry and amplitude was comprised of 450–750 stimulus pulses distributed across individual continuous trials for each setting and aligned with the stimulus pulses using custom scripts in MATLAB 2021a (Mathworks, Natick, MA, United States). For the CPL geometry, the electrophysiology data for all analyses were aligned to the second, larger amplitude phase of the stimulus pulse (φ), as the conditioning pulse (ω) was subthreshold for eliciting a measurable response at such small amplitudes and large pulse widths. For scalp EEG data, the grand average of all EEG channels was subtracted from each individual EEG channel to remove common noise. A 10-point moving average filter to remove high frequency noise and 0.5 ms baseline subtraction was applied to remove the DC shift after averaging (16, 18, 20).

#### EMG measurements

The MEP was derived using the averaging process described above for the EEG. The response period from 10 to 50 ms was compared to the baseline period prior to the artifact from −50to 0 ms to determine whether an MEP response was present and rising above the noise floor and residual activity in the EMG. The standard deviation of the response period needed to exceed 4× the standard deviation of the baseline period for further quantification of the MEP. Only participants and channels with at least one MEP response above that threshold in the SB− condition were included for analysis regardless of whether a channel showed an MEP response above threshold in another geometric condition. We did not observe consistent responses in other conditions from participants with no response in the SB− condition. After thresholding for inclusion the MEP response was quantified using the root mean square (RMS) from the 10 to 50 ms period (39). The RMS was normalized for group analysis (across participants) using the following formula.


$$x_{norm}=\frac{\left(x_{i}-x_{\min}\right)}{\left(x_{\max}-x_{\min}\right)}$$

Where x_norm_ = the resulting normalized value ranging from 0 to 1, x_i_ = the RMS of an individual MEP response, x_min_ = the minimum RMS value of an individual MEP response from a participant’s dataset, x_max_ = the maximum RMS value of an individual MEP response from a participant’s dataset. This normalization allowed for responses to be shown as a percent of the maximal observation (values ranging from 0 to 100%) across all tested pulse geometries.

#### EEG short-latency components

After visual inspection of the current source density (CSD) plots for the SB− geometry it was determined that the response with the highest amplitude was localized in electrodes C4, FC2, Cz, and CP2 across studies patients. To robustly capture the responses and compare them between patients, we averaged these four electrodes and used the average as a single channel to quantify the short latency components of the EEG response. Visualization of the CSD plots were performed using the Brainstorm toolbox in MATLAB (40). To characterize the short-latency components (<7.7 ms) the amplitude of the first observable positive peak (~2 ms) following the onset of stimulation was measured relative to the following negative deflection (i.e., peak-to-peak amplitude). This peak is consistent with prior studies, where it has been argued to represent antidromic activation of the hyper-direct pathway between the primary motor cortex and STN (20, 41). Due to the prolonged, secondary phase in the two CPF conditions, the electrical artifact extended up to 4 ms after the onset of stimulation and precluded identification and quantification of these short-latency components. As such, short-latency peak-to-peak measurements were not made for those conditions. We did not attempt to remove or blank the artifact prior to quantification of the short-latency components, consistent with prior publications reporting on this component (20, 42).

#### EEG long-latency components

**Figure 2:**
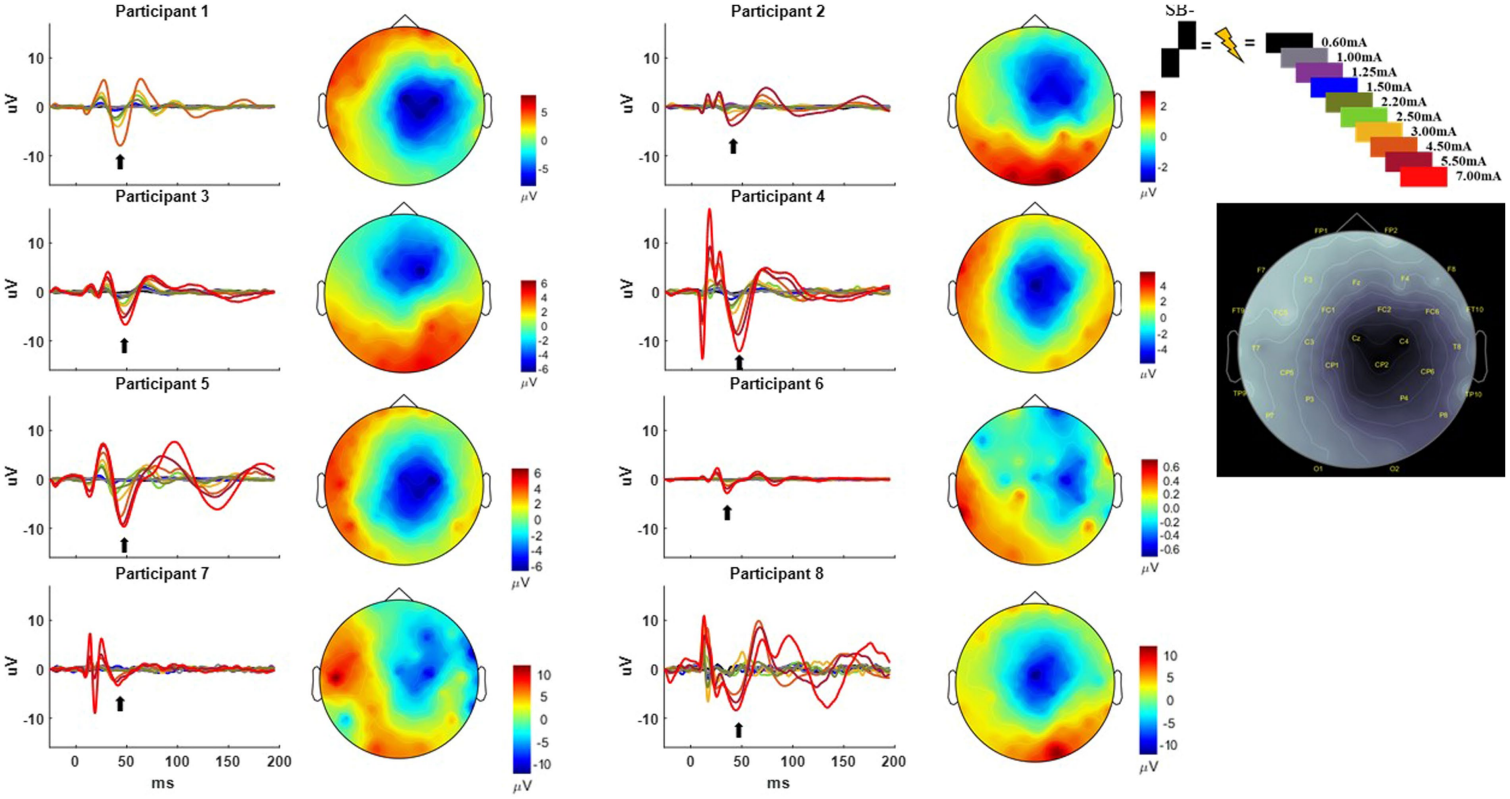
Time-domain response from each participant averaged across the C4, FC2, Cz, and CP2 electrodes using the SB− pulse geometry. Color key for the amplitude of stimulation is shown on the upper right from 0.60 to 7.00 mA. Current source density plot aligned to ~50 ms (with arrow indicating the timepoint from which the CSD is derived) is shown adjacent to each participant’s time domain response with the uV scale bar. Electrode locations with their corresponding labels generated via Brainstorm are shown on a gray scale replica of the response from Participant 1 on the far right.

The long-latency components were measured across the same four electrodes averaged together to a single channel as was done for the short-latency components. The full time course of the single channel average and the current source density plots aligned to the 50 ms time point can be seen in Figure 2. The artifact was blanked for 5 ms and the DBS-CEP was zero padded to allow for an 8-100 Hz band pass filter to remove additional noise. We used the continuous wavelet transform (CWT) function in MATLAB utilizing the analytic Morse wavelet with 60 cycles to estimate the amplitude of the cortical DBS-EPs as a function of frequency and time (18, 43–45). We utilized the time-frequency decomposition to evaluate the long-latency components of the CEP and quantify them for group comparison and analysis. To measure wavelet amplitudes for group analysis, the average wavelet amplitude was calculated in the beta and gamma frequency bands. We averaged the wavelet amplitude in the 13–35 Hz (beta) band from 25 to 75 ms, and within the 36–100 Hz (gamma) band from 5 to 50 ms. These regions for both the beta and gamma frequency bands were selected based on a visual inspection of the wavelet spectrograms at each amplitude to identify the time points where the spectrograms exhibited their highest amplitudes. The minimum and maximum wavelet amplitudes of the evoked responses for a given participant across all stimulation amplitudes and pulse geometries were used to normalize their responses for group analysis (across patients). The same normalization formula used above for the EMG measurements was applied here to the EEG data. This normalization allowed for the responses to be shown as a percent of the maximal observation observed across all tested pulse geometries.

#### Statistics

Generalized linear mixed effects models (GLMM) were used to quantify the increase in percent activation of myogenic and cortical activity as a function of increasing stimulation amplitude, separately for each pulse geometry and stratified by polarity (mono- vs. bipolar), frequency band, and recording location (46). GLMM are flexible extensions of linear regression that can account for multiple observations per subject and non-Gaussian response data. Each model included a random effect for subject, a fixed effect for amplitude (continuous) and a compound symmetry covariance structure. Multivariate distribution was investigated visually using Chi-squared quantile by squared Mahalanobis Distance plots and selected distributions were confirmed via examination of model residual panel and Bayes Information criterion. For cortical beta, cortical gamma, and MEPs, data were multivariate lognormal and results are presented as exponential slopes to indicate the exponential increase in response for every one-unit increase in stimulation amplitude for each pulse geometry. For the short-latency peak-to-peak measurements, data were multivariate normal and are presented as linear slopes to indicate the linear increase in response for every one-unit increase in stimulation amplitude for each pulse geometry. Estimates were generated using restricted maximum likelihood.

95% confidence intervals (CI) were generated for each slope. CI for the slopes of each of the six geometries were compared to one another, separately within each polarity and within each region/frequency (i.e., six slopes and CI for monopolar configurations within cortical beta, six slopes for bipolar configurations within cortical beta, etc.). If two CI did not overlap, this was considered to be indicative of a statistically significant difference in the two slopes. When this occurred, GLMM were used for pairwise comparisons: model specifications were as previously described, but with two geometries included, along with an interaction term for geometry by amplitude. This interaction was the coefficient of interest, to determine whether percent activation increased significantly more as a function of amplitude for one geometry compared to another.

Lastly, to identify the amplitude at which percent activation was significantly different from zero, a series of GLMM were used as first described (one model per geometry and stratified by polarity and region/frequency), but with tested amplitude values as a categorical variable instead of continuous. Percent activation for each categorical amplitude tested was compared with zero, and the lowest amplitude with activation significantly different from zero is presented. Due to the exploratory, hypothesis-generating nature of the study, correction for multiple comparisons was not made (47). Analyses were completed using SAS Studio v 3.81 (SAS Cary, NC, United States).

## Results

### Participant demographics and DBS lead location

Data were collected from eight participants (3 female), with a mean age and disease duration of 63.8 ± 2.5 years and 6.7 ± 2.4 years, respectively. The average pre-operative, OFF-medication, total MDS-UPDRS-III score was 41.4 ± 21.54. The average post-operative, OFF-medication, total MDS-UPDRS-III was 33.5 ± 20.44, indicating that the lead implantation alone was associated with an improvement of 19 % in the UPDRS-III (Table 1). The DBS leads had two to three contact rows implanted within the posterior part of STN (Figure 1A). All contacts selected for stimulation via the monopolar review were estimated to be inside the posterior STN based on pre-operative MRI and post-operative CT image co-registration. There were no complications from surgery or during testing.

**Table 1:** Participant demographics.

Participant	Age (years)	Disease duration (years)	Sex	Implant side	Off medication preoperative UPDRS-III score	Off medication postoperative UPDRS-III score	Relative change (percentage)	Preoperative levodopa equivalent daily dose	Position of stimulation contact [from midcommisural point (mm)]			Lead type	Lead manufacturer
									X	Y	Z		
1	62.5	4	Female	Right	33	46	+13 (39.4%)	1,043	11.1	−1.1	0.5	B33005	Medtronic
2	68.4	11	Female	Left	25	14	−11 (56.0%)	1,030	−11.3	−1.2	1.1	B33005	Medtronic
3	64.7	5	Male	Right	52	45	−7(13.5%)	1,010	12.6	−2	2.6	B33005	Medtronic
4	65.9	8	Male	Right	41	40	−1(2.4%)	800	12.1	−2.6	2.6	6,172	St. Jude
5	62.3	5	Male	Right	74	70	−4(5.4%)	1,342	11.5	−1.5	5.2	B33005	Medtronic
6	65.5	11	Male	Left	43	25	−18(41.8%)	1,110	−10.7	−2.97	2.2	B33005	Medtronic
7	60.2	9	Male	Right	14	19	+5(35.7%)	1,275	11.89	−4.11	5.3	B33005	Medtronic
8	62.7	5	Female	Right	35	9	−26 (74%)	1,825	13.03	−3.29	3.5	B33005	Medtronic

### MEP response

**Figure 3:**
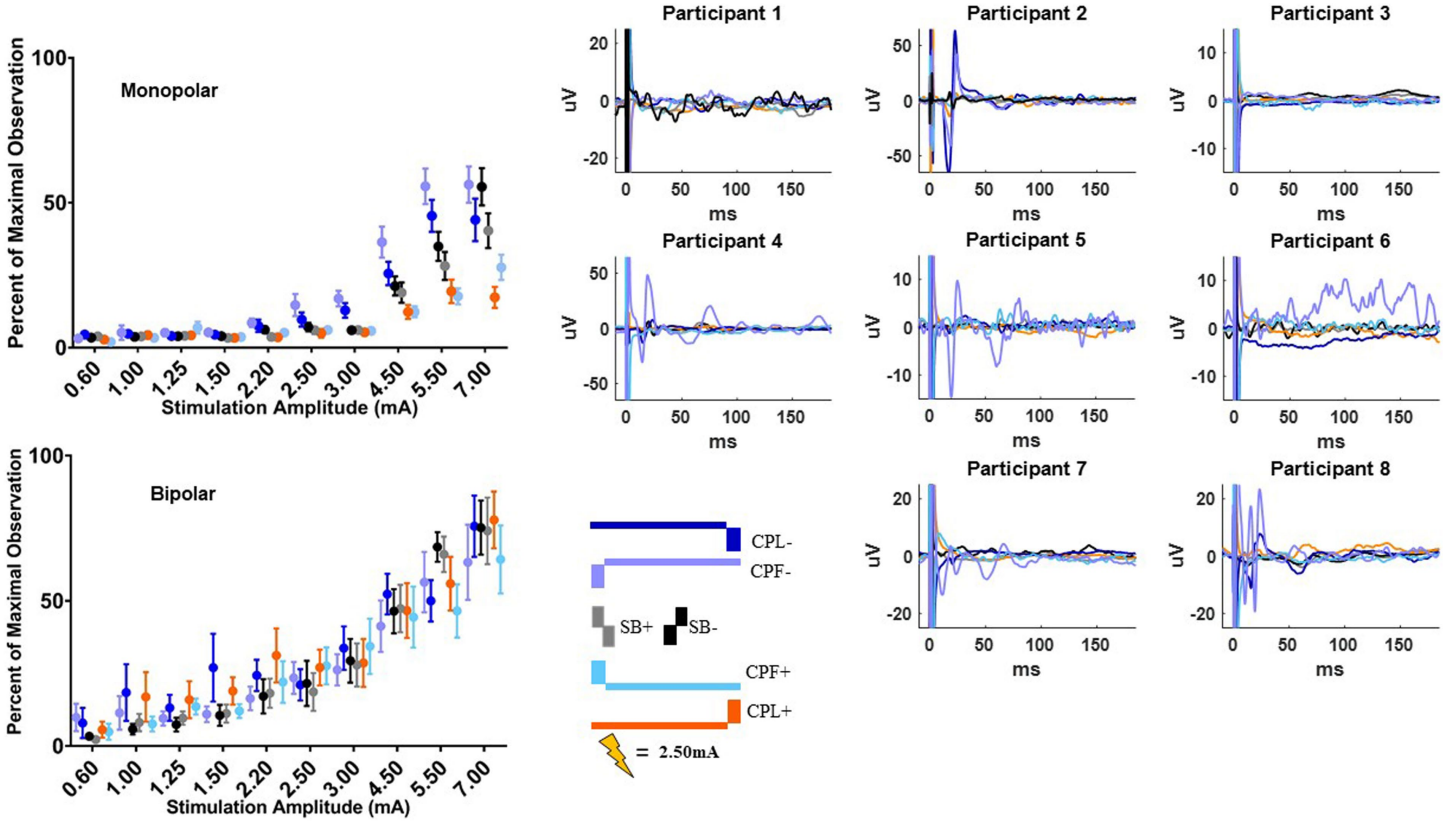
MEP individual and group results for all six pulse geometries. Each individual participant’s response across the six pulse geometries is shown on the six plots on the right from the monopolar configuration. The amplitude is fixed at 2.50 mA for each geometry. The normalized RMS measurement averages +/− SEM are shown on the upper left from the monopolar configuration. The bottom left plot shows the normalized RMS in the bipolar configuration.

The MEP responses and group results from manipulating pulse geometry can be seen in Figure 3. Two out of eight participants (1 and 3) did not show any MEP response to monopolar stimulation (up to 7.0 mA) across the electrodes placed on the arm contralateral to stimulation and were excluded based on the MEP thresholds described above. For the patients who exhibited a supra-threshold MEP response in the monopolar montage, a clear biphasic response can be observed around 25 ms. The response was largest in the time-domain trace across the CPL− and CPF− conditions. The CPL+ and CPF+ conditions were associated with the smallest MEP response. Group results of the quantified RMS from 10 to 50 ms normalized to the baseline at the end of the response profile can be seen on the left side of Figure 3. These group results demonstrate a shift in thresholds for MEP responses as a function of changing pulse geometry.

For monopolar configurations, activation as a function of amplitude increased more rapidly for CPF-, when compared to all other geometries (*p* < 0.0001 when compared to CPF+ and CPL+; *p* = 0.0006 when compared to SB+; *p* = 0.040 when compared to CPL−; and *p* = 0.041 when compared to SB−); for CPL−, when compared to CPF+ (*p* = 0.002) and CPL+ (*p* < 0.0001); and for SB−, when compared to CPF+ (*p* = 0.001) and CPL+ (*p* < 0.0001). Specifically, activation increased by 55% (95% CI 49–60) per mA for CPF− (all *p* < 0.0001). The CPL− condition increased by 47% (95% CI 41–52) per mA. SB− increased by 46% (95% CI 41–52) per mA, while SB+ increased by similarly by 41% (95% CI 35–46). The CPF+ increased by 33% (95% CI 27–39) per mA and CPL+ increased by 28% (95% CI 23–34) per mA.

No differences in the EMG response as a function of pulse geometry were observed in the bipolar montage. GLMM slopes and their confidence intervals can be found in Table 2. The amplitude at which the response became significantly different from zero can be found in Table 3.

**Table 2:** Statistical findings comparing the differences in slope from a generalized linear mixed effect models as a function of changing pulse geometry.

Geometry	MEP			Antidromic peak			Cortical-EP beta band			Cortical-EP gamma band		
	Slope	LCL	UCL	Slope	LCL	UCL	Slope	LCL	UCL	Slope	LCL	UCL
**Monopolar**												
CPF+	0.33	0.27	0.39				0.43	0.35	0.52	0.4	0.31	0.48
CPF−	0.55	0.49	0.6				0.57	0.48	0.65	0.48	0.38	0.59
CPL+	0.28	0.23	0.34	0.03	0.01	0.04	0.55	0.44	0.67	0.58	0.48	0.69
CPL−	0.47	0.41	0.52	0.11	0.09	0.13	0.58	0.5	0.66	0.57	0.49	0.66
SB+	0.41	0.35	0.46	0.1	0.09	0.11	0.69	0.57	0.81	0.6	0.51	0.69
SB−	0.46	0.41	0.52	0.13	0.11	0.15	0.59	0.51	0.68	0.58	0.49	0.67
**Bipolar**												
CPF+	0.13	0.07	0.18				0.44	0.35	0.54	0.31	0.24	0.39
CPF−	0.03	−0.02	0.09				0.44	0.34	0.55	0.33	0.24	0.42
CPL+	0.05	−0.005	0.11	0.04	0.02	0.07	0.47	0.36	0.57	0.42	0.32	0.52
CPL−	0.12	0.06	0.17	0.08	0.06	0.11	0.42	0.31	0.53	0.38	0.3	0.47
SB+	0.13	0.08	0.17	0.09	0.07	0.11	0.56	0.47	0.65	0.47	0.36	0.58
SB−	0.12	0.06	0.17	0.12	0.1	0.14	0.57	0.46	0.62	0.44	0.35	0.52

Slopes for each pulse geometry across the four measurements (MEPs, are indicated in the “slope” column). Their corresponding lower confidence limit (LCL) and upper confidence limit (UCL) are adjacent. For example, the MEP confidence interval for CPF+ or 0.27–0.39 does not overlap with the MEP confidence interval for CPF− or 0.49–0.6, indicating the two slopes are significantly different. Where confidence intervals do not overlap, that indicates that the slopes are significantly different. These significant differences are highlighted in blue and yellow. Confidence limits with blue and yellow highlight in split cells reflect slopes that are significantly steeper than at least one other slope and are significantly less steep than at least one other slope from another pulse geometry at that recording location within that montage. ^*^Electrical artifact precluded measurement of the CPF+/− condition in this metric. The color coding under the “Geometry” column in table reflects the corresponding pulse geometry color coding from Figure 1.

**Table 3:** Statistical findings presenting the lowest amplitude where percent activation began to be significantly different from zero.

Geometry	MEP	Antidromic peak	Cortical-EP beta band	Cortical-EP gamma band
**Monopolar**				
CPF+	4.5		4.5	3
CPF−	3		2.2	2.2
CPL+	4.5	4.5	4.5	4.5
CPL−	4.5	2.2	2.2	2.2
SB+	4.5	2.2	2.5	2.5
SB−	4.5	2.2	2.2	2.2
**Bipolar**				
CPF+	1.25		2.2	1.25
CPF−	0.6		2.2	2.2
CPL+	0.6	4.5	1.5	1.5
CPL−	0.6	3	1.5	2.2
SB+	0.6	4.5	2.2	1.5
SB−	0.6	3	2.2	1.25

In the monopolar CPL+ condition the amplitude was only different at 4.5 mA, but not 5.5 or 7.0 mA for the short-latency CEP. ^*^Electrical artifact precluded measurement of the CPF+/− condition in this metric. The color coding under the “Geometry” column in table reflects the corresponding pulse geometry color coding from Figure 1.

### Short-latency cortical response

**Figure 4:**
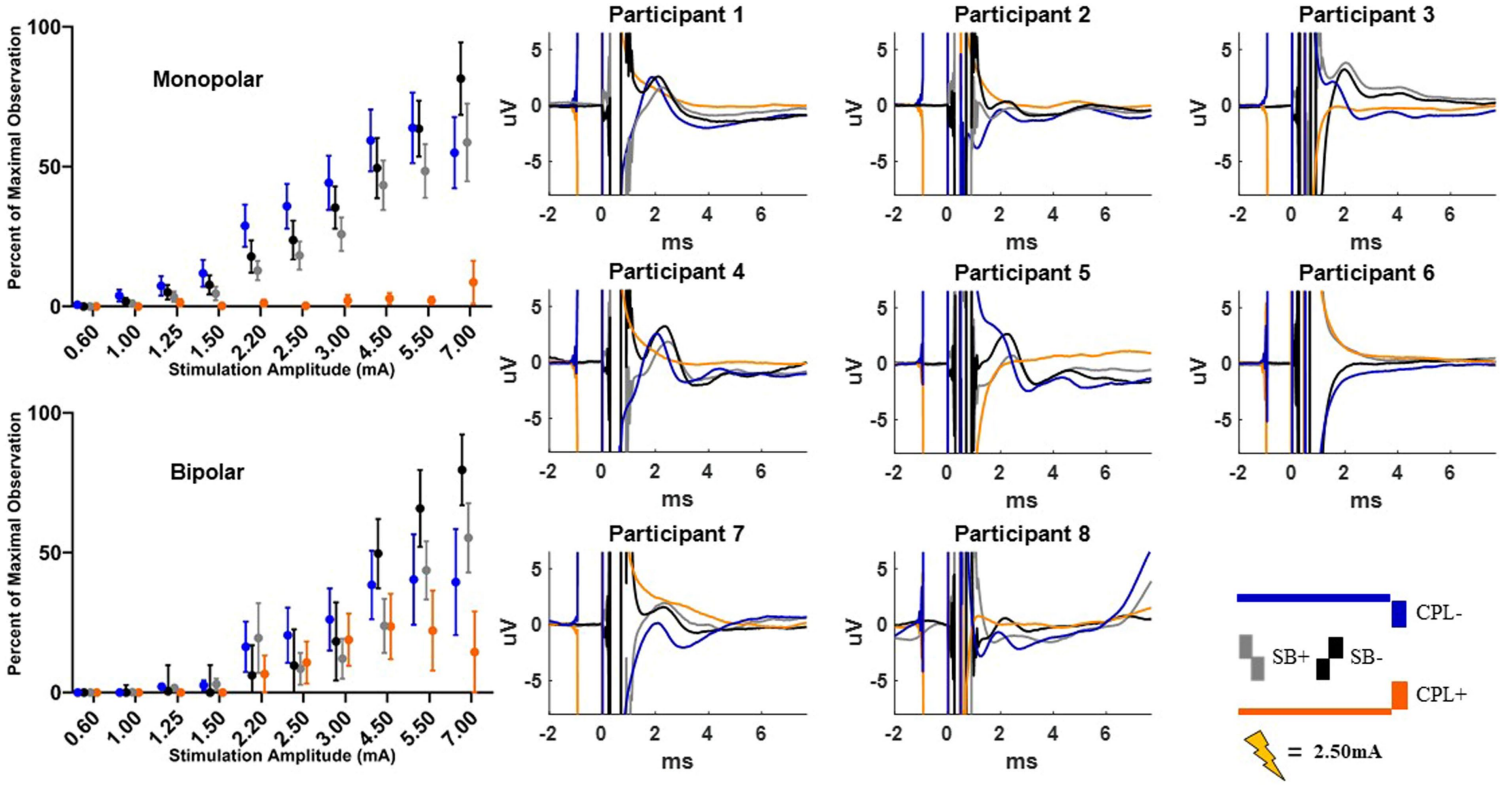
Short latency DBS-CEP peaks and quantified group results. Each individual participant’s response across the four pulse geometries is shown on the eight plots on the right from the monopolar configuration. The amplitude is fixed at 2.50 mA for each geometry. The normalized peak to peak amplitude measurements (average +/−SEM) are shown on the upper left from the monopolar configuration. The bottom left plot shows the normalized peak to peak measurements (average +/−SEM) from the bipolar configuration.

Short-latency components (<7.7 ms) could be discerned for four out of six pulse geometries (SB−, SB+, CPL−, and CPL+) in both the monopolar and bipolar montages. We were unable to reliably measure short-latency components with the CPF− and CPF+ geometries due to artifact contamination. The data for the peak-to-peak measurements of short-latency components best fit a linear model. A discernable peak began to emerge around 2 ms that was measured relative to the negative deflection that followed (see Figure 4). In the monopolar montage, the response for the CPL+ condition in participants 3 and 8 could only be identified once the amplitude of stimulation reached 3.0 mA and 1.5 mA, respectively. In the remaining participants, no short-latency peak was observed in the CPL+ condition regardless of amplitude. A short-latency peak could be observed as low as 1.00 mA in the CPL−, SB+, and SB− condition. For monopolar configurations, activation as a function of amplitude increased more rapidly for CPL− (*p* < 0.0001), SB+ (*p* < 0.0001), and SB− (*p* < 0.0001), when compared to CPL+. See Table 2 for the slopes and confidence intervals. The growth in the response curve was largest for the pulse geometries hypothesized to preferentially recruit axons (i.e., CPL−) compared to those hypothesized to preferentially recruit cell bodies (i.e., CPL+). For monopolar configurations, activation increased by 0.03 uV (95% CI 0.01–0.04) per mA for CPL+, which was significantly less than the 0.10 uV (95% CI 0.09–0.11, *p* < 0.0001) increase per mA for SB+, the 0.11 uV (95% CI 0.09–0.13, *p* < 0.0001), increase per mA for CPL− and the 0.13 uV (95% CI 0.11–0.15, *p* < 0.0001) increase per mA for SB−. The threshold at which the response was significantly greater from zero can be found in Table 3. The CPL+ condition was only significantly different from zero at 4.5 mA, while the others were significant at the listed amplitudes and all amplitudes above.

When using a bipolar montage, there was no short-latency response below 1.25 mA for all pulse geometries and a minimal response thereafter until 2.20 mA. Thereafter, the growth in the response curve was again largest for the axon recruiting pulse geometries (i.e., CPL−) compared to those hypothesized to preferentially recruit cell bodies (i.e., CPL+). When comparing the slope of the curve for each geometry, activation as a function of amplitude increased more rapidly for SB−, when compared to CPL+ (*p* < 0.0001). For bipolar configurations, SB− increased by 0.12 units (95% CI 0.10–0.14) per mA, which was significantly greater than the 0.04 units (95% CI 0.02–0.07, *p* < 0.0001) increase for CPL+. Overall, the effect of pulse geometry on the short-latency components was less robust in the bipolar montage compared to the monopolar montage.

### Long-latency cortical response

**Figure 5:**
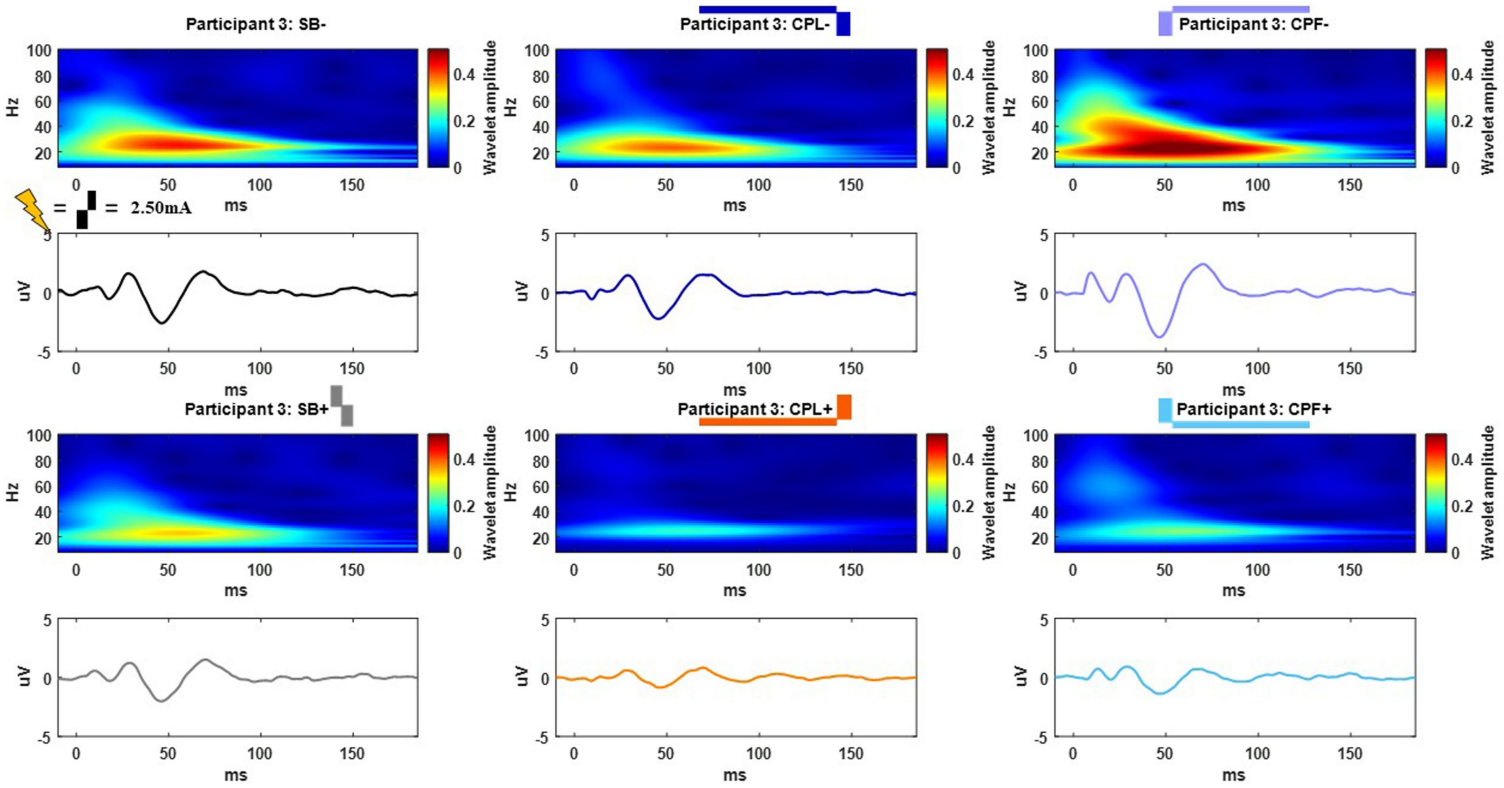
Example time and frequency domain response (average of Cz, FC2, C4, CP2 electrodes) of the DBS-CEPs and for each pulse geometry at 2.50 mA from Participant 3. The pulse geometry reflecting each response is labeled above the spectrogram (stimulus waveform not to scale).

The long-latency components were quantified using the wavelet transform. An example time-domain trace and corresponding spectrogram for each pulse geometry at 2.50 mA is shown in Figure 5 for the monopolar montage. The time domain trace shows a series of positive and negative deflections from 10 to 150 ms after stimulation onset that differ as a function of pulse geometry (see Figure 5). Spectrograms showing the frequency content of the averaged evoked response with respect to time reveal that high-amplitude components were concentrated in the beta band (13–35 Hz), which is consistent with prior studies (18, 21). This amplitude component is observed between 25 and 75 ms and maximal at 50 ms. The amplitude of the wavelet response in the beta band was largest in the cathode-oriented pulse geometries (SB−, CPL−, and CPF−), with the anodic pulse geometries having resulted in evoked responses with lower amplitudes. The spectrogram also showed elevated gamma band (36-100 Hz) activity that occurs between 0 and 50 ms. This gamma component was most robust in the CPF− condition, however, it was also evident across other pulse geometries.

**Figure 6:**
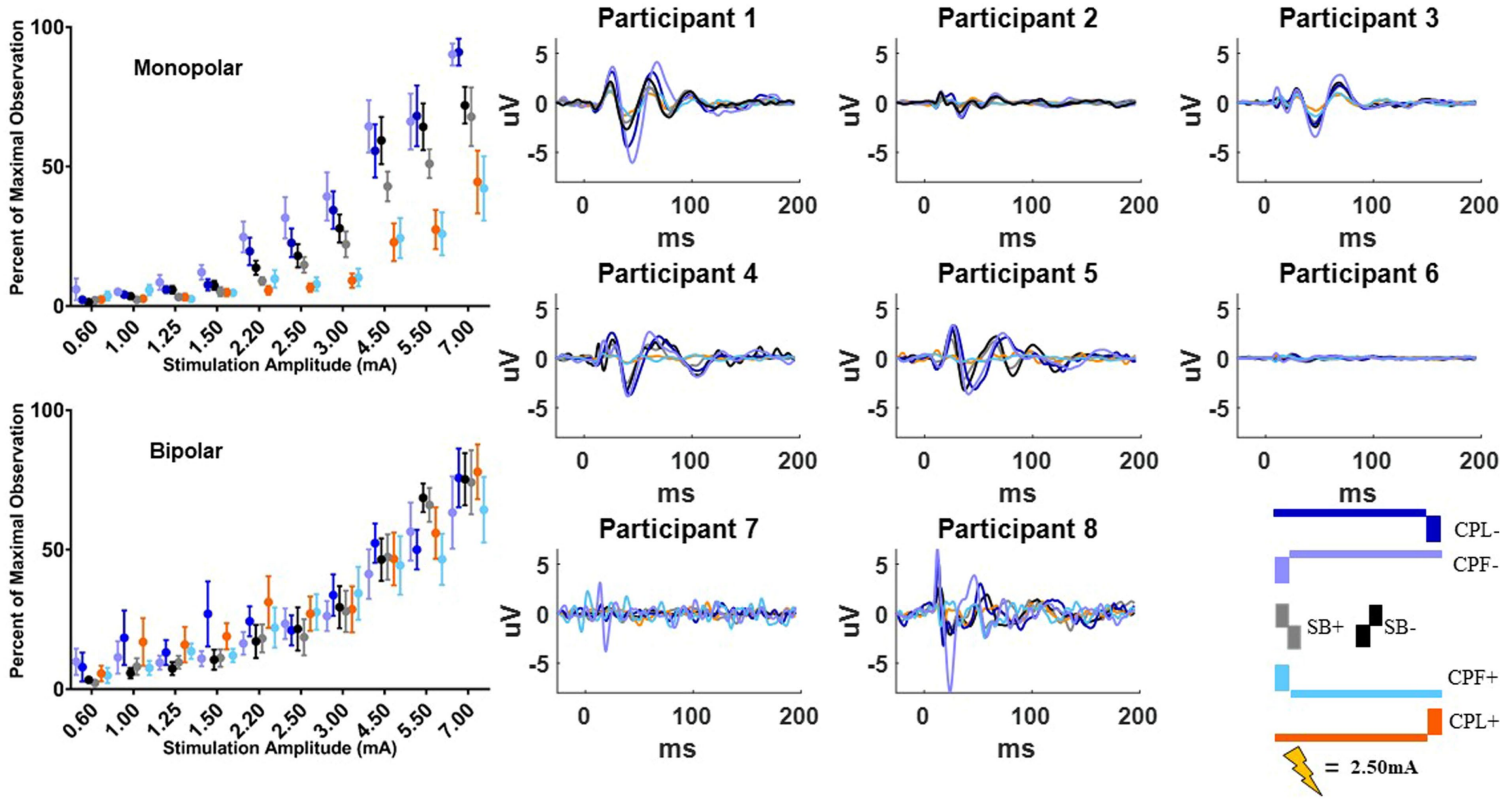
Long-latency cortical beta (13–35 Hz) band individual and group results for all six pulse geometries from −10 to 200 ms aligned to the stimulus pulse. Each individual participant’s response across the six pulse geometries is shown on the eight plots on the right from the monopolar configuration. The stimulus amplitude generating the time domain plots is fixed at 2.50 mA for each pulse geometry. The normalized averaged wavelet amplitude measurements in the beta (13–35 Hz) band from 25 to 75 ms are shown on the upper left from the monopolar configuration. The bottom left plot shows the normalized averaged wavelet amplitude from the beta band in the bipolar configuration. Error bars reflect standard error of the mean.

The group results are quantified in Figure 6 and shown for both the monopolar and bipolar montages. When comparing the time-domain trace the peak morphology shows either a positive or negative deflection or phase shift in the components at approximately 25 and 50 ms, respectively, in most participants as a function of pulse geometry. The overall amplitude of the time-domain trace is attenuated in the CPL+ and CPF+ conditions compared to the CPL− and CPF− conditions.

The quantified response using the average maximum wavelet amplitude for the beta frequency content for group comparison is shown on the left side of Figure 6. In the monopolar montage the response curve is led by the CPF− condition, followed by CPL−, SB−, SB+, CPL+, and CPF+. The wavelet amplitude changes as a function of stimulus amplitude and increased more rapidly for SB+, when compared to CPF+ (*p* = 0.001). This response could be appreciated at amplitudes as low as 0.60 mA for the CPF− condition. For monopolar configurations, activation increased by 69% (95% CI 57–81) per mA for SB+, but only 43% (95% CI 35–52) per mA for CPF+ (p = 0.001; no other significant differences between geometries in the monopolar configuration). Of note, there were no differences in the response profiles as a function of pulse geometry when stimulation was delivered using the bipolar configuration. The slope and corresponding confidence intervals for the exponential slope fitting these data can be found in Table 2. The amplitude at which the response was significantly different from zero can be found in Table 3.

**Figure 7:**
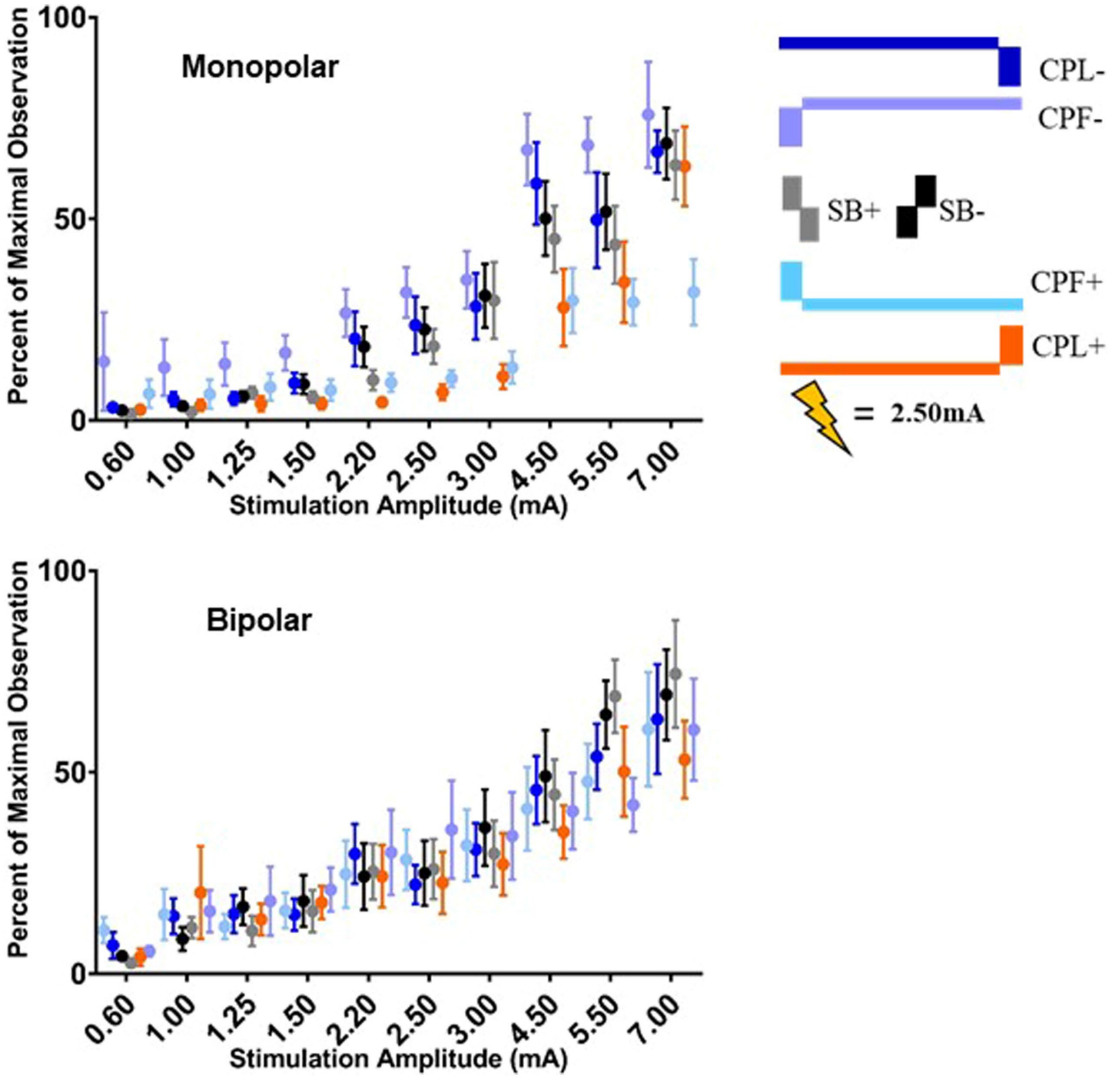
Long-latency cortical gamma (36–100 Hz) band group results for all six pulse geometries quantified from 5 to 50 ms. The normalized averaged wavelet amplitude measurements in the gamma band are shown on the upper panel for the monopolar configuration. The bottom panel shows the normalized averaged wavelet amplitude in the gamma band from the bipolar configuration. Error bars reflect standard error of the mean.

The quantified group results for the gamma band, using the average maximum wavelet amplitude, are shown in Figure 7. For monopolar configurations, activation as a function of amplitude increased more rapidly for CPL− (*p* = 0.013), SB+ (*p* = 0.003), and SB− (*p* = 0.008), when compared to CPF+. For monopolar configurations, activation increased by 40% (95% CI 31–48) per mA for CPF+, which was significantly less than the 57% (95% CI 49–66, *p* = 0.013) increase per mA for CPL−, the 58% increase per mA for SB− (95% CI 49–67, *p* = 0.008), and the 60% (95% CI 51–69, *p* = 0.003) increase per mA for SB+. There were no other significant differences between geometries. Similar to the beta frequency content, the responses begin to diverge for pulse amplitudes of 1.25 mA and higher and the bipolar montage showed no differences as a function of pulse geometry. The gamma band long-latency responses also best fit an exponential model and the values can be found in Table 2 along with the amplitudes where the response became significantly different from zero in Table 3.

## Discussion

This study provides *in vivo* evidence that manipulating pulse geometry can have a significant impact on the physiological effects of STN DBS. This effect was most robust for monopolar stimulation and largely absent in the bipolar condition, with the exception of the short-latency components of the DBS-CEP. The CPF− waveform, which most closely emulates the waveform used in FDA-cleared IPGs in the US, showed the lowest MEP activation threshold. Notably, the next lowest was from the CPL− waveform, which has been hypothesized to be preferential for recruiting axons (6). When the primary, large-amplitude phase was anodic (CPL+ and CPF+) the threshold for MEP activation increased in the monopolar configuration. Similarly, the short and long-latency components of the DBS-CEPs response to CPL+, hypothesized to be preferential for recruiting cell bodies (6), were attenuated relative to the CPL− condition for both the short and long-latency responses. These results suggest that manipulating pulse geometry may allow for enhanced neural selectivity and, as a result, potentially impact the upper bounds of the therapeutic window of STN-DBS.

### DBS-CEP and -MEP findings support computational modeling-based predictions

Charge balancing is critical to ensure safe delivery of electrical stimulation for *in vivo* use (48). However, charged balanced symmetric biphasic pulses (i.e., SB+/−) minimize any neural selectivity for cell bodies vs. axons (6). McIntyre and Grill (6) introduced the idea of using asymmetric charge balanced pulses (i.e., CPL+/−) in DBS to restore the neural selectivity in extracellular stimulation that is observed when using monophasic pulses (6). This built upon prior, primarily *in-vitro*, work demonstrating that a reduction of pulse amplitude and increased pulse width of the first phase of stimulation allows for the exploitation of non-linear conductance properties of H and M gate dynamics in sodium channels (8, 49–52). Changing the H and M gate dynamics ultimately alters the excitability of the neuronal membrane and its response to the second phase of the stimulus pulse—referred to as the primary phase in the current study.

Our study results align with the modeling work of McIntyre and Grill (6) in two ways. First, we demonstrated differences in the response curve through the use of varying types of asymmetric biphasic pulses. The differences that we observed may reflect the enhanced selectivity of cell bodies vs. axons when employing geometric manipulation to the stimulus pulse. However, the ability to explicitly conclude cell body vs. axon activation is beyond the scope of the data reported here and deserve further research in a preclinical model where such differentiation is possible. Second, we showed that this potential selectivity is greatest when utilizing a monopolar configuration. This may be because a bipolar stimulation montage negates selective exploitation of sodium channel dynamics via the hyperpolarization or depolarizing conditioning phase by placing the inverse effect in closer spatial proximity than is achieved with a “monopolar” montage that uses the metal casing of patient’s implantable pulse generator located at the level of the chest as the return electrode. The use of a bipolar montage also eliminates any effects of polarity, therefore a bipolar montage is not truly anodic vs. cathodic when applied across adjacent contacts as done in this study and may explain why no difference was observed in a bipolar montage. Future work should explore the distance threshold between contacts in a bipolar montage that is necessary in order to observe effects from changing pulse geometry. Overall, these findings demonstrate that introducing an extended depolarizing or hyperpolarizing conditioning phase influences the stimulus selectivity. This selectivity was evident in both the MEP and DBS-CEP data.

### Decreased motor side effects through anodic pulse geometries

One of the most common side effects of STN DBS is undesirable current spread to the corticospinal tract resulting in muscle twitches and pulling, which can be measured using MEPs (10–13). In this study we demonstrate the threshold for eliciting an MEP response is higher when using the pulse geometries where the primary phase is anodic (i.e., CPL+ and CPF+). This parallels prior findings (7, 53) that suggest the therapeutic window was greater in STN-DBS when employing anodic compared to cathodic stimulation using pulse geometries similar to CPF+/− from this study. Taken together these results suggest that the ability to preferentially target neural components in the region of the STN that avoid undesirable capsule activation is possible with pulse geometries where the primary phase is anodic. However, this study evaluated a subset of muscles on the arm contralateral to stimulation. Further work would benefit from exploring additional muscles as well as the differing effects that anodic stimulation can produce on distinct neuronal elements (i.e., dendrites, soma, and axons) in the STN and neighboring regions compared to cathodic stimulation in PD to better understand the mechanisms behind reduced capsule activation.

### Hyperdirect pathway activation with cathodic but not anodic stimulation

The hyperdirect pathway is comprised of layer 5 pyramidal neurons with axonal projections that innervate the STN (25). Prior work has shown that antidromic activation of this pathway appearing within the first 7 ms from the onset of stimulation is present during clinically effective STN-DBS and may be involved in the therapeutic mechanism of STN-DBS (20, 26–28, 41). Modeling studies further support the feasibility of peaks occurring within the first 7 ms as being of physiological origin and possibly attributable to antidromic activation from STN stimulation (29, 30, 38, 54). Results from this study demonstrate that a peak can be observed as early as ~2 ms post the onset of stimulation that changes as a function of manipulating pulse geometry and may be reflective of antidromic activation of the hyperdirect pathway. This peak presented only with the CPL−, SB−, and SB+ pulse geometries for the monopolar configuration. The anodic pulse geometry (CPL+) showed an absence of a peak at ~2 ms except for an amplitude of 4.5 mA during monopolar stimulation, despite previous modeling work that suggests anodic pulse geometries (such as CPL+) may preferentially activate the terminating axons in the hyperdirect pathway and more readily produce antidromic activation (29, 30). Future studies are needed to assess whether the CPL+ pulse geometry is capable of producing therapeutic benefit when delivered at high frequency in the absence of antidromic activation to clarify further the role of the hypderdirect pathway (antidromic) activation in the mechanism of STN-DBS.

### Long-latency components and orthodromic pathways

The long-latency components in the DBS-CEPs have been tied to postoperative motor side effects, disease severity, and the fluctuation of dopamine due to medication cycling (11, 21, 23, 42, 55, 56). The impact on the long-latency components of manipulating pulse geometry was examined through the changes in wavelet amplitude in both the beta and gamma bands. Our results show that the changes were exclusive to monopolar stimulation, which is consistent with the original modeling work by McIntyre and Grill (6). Other previous modeling studies have proposed that the long-latency response can occur without additional orthodromic activation of the cortex (57). However, results from this study show that the long-latency response in the CPL+ condition occurred at low stimulation amplitudes, while the short-latency peaks did not emerge until stimulation reached a higher amplitude, which suggests orthodromic pathways may be contributing to the long-latency cortical response. This is further supported by the reduced or absent MEP response in the CPL+ condition, which suggests that the long-latency response was likely not due to activation of the corticospinal tract fibers independent of the hyperdirect pathway. Both the short and long-latency cortical responses emerged prior to the MEP responses, which may also suggest that CEP thresholds may relate more to benefit than side effects.

### Manipulating pulse geometry and clinical use

Many preclinical and clinical studies have evaluated DBS using a symmetric biphasic pulse geometry despite their absence in the clinical IPGs providing therapeutic stimulation in implanted patients (4, 5). Given the differences demonstrated here and in prior clinical (7, 53, 58–60) and modeling (29) work, a lack of consideration for pulse geometry in study designs may confound efforts toward clinical translation. The results shown here suggest that changes in pulse geometry are exclusive to monopolar stimulation, which is often the clinical standard due to yielding a larger volume of tissue activation and necessitating lower current levels to solicit therapeutic benefit (61–63). In this study we observed reduced motor evoked potentials when utilizing the pulse geometry consisting of an anodic pre-pulse and cathodic primary pulse (CPL−) while maintaining the CEP. If the CEP is related to the therapeutic benefit of STN-DBS as previous groups have suggested, it would indicate that the CPL− pulse geometry may provide a better therapeutic window compared to conventional DBS waveforms where a low amplitude anodic phase follows the cathodic pulse.

Manipulating pulse geometry may best serve PD patients with poor symptom control or low side-effect thresholds where traditional stimulation approaches produce poor outcomes. Future studies would benefit from evaluating the behavioral effects of manipulating pulse geometry in those specific patient populations and may serve as an additional option prior to surgical interventions to reposition the DBS lead. Prior work by Anderson et al. (64) demonstrated that lead position and the polarity of stimulation also influences the fibers activated during DBS, which may influence the variability in responses to changes in pulse geometry. Through modeling they demonstrate that fibers running orthogonally to the electrode are more easily activated by anodic stimulation at lower thresholds than fibers of passage are through cathodic stimulation. The results reported here combined with the findings from Anderson et al. (64) suggest that patients may benefit from DBS devices that allow for manipulating pulse geometries across different contacts to facilitate targeting of specific neural pathways that may be associated with control of different motor symptoms of PD. Future research should explore the physiological and behavioral effects of evaluating pulse geometry spatially across directional contacts on the DBS lead.

## Conclusion

The results from this study provide physiological evidence that pulse geometry may afford enhanced neural selectivity, with potential clinical implications related to modifying the DBS therapeutic window—specifically the side effect profile. Pulse configurations that target axons show increased capsule recruitment, while those targeting cell bodies show higher thresholds for comparable capsule recruitment. Cortical evoked potentials show morphological and frequency changes as a function of pulse geometry that demonstrate how enhancing neural selectivity may target different pathways in the basal-ganglia thalamocortical circuit. The specific impact of changing pulse geometry on the short-latency cortical responses possibly reflecting antidromic activation of the hyperdirect pathway suggest that manipulating pulse geometry may also provide an additional tool in investigating potential mechanisms of DBS through selective targeting of proposed mechanistic pathways in the basal-ganglia thalamocortical circuit.

### Limitations

Participants were not asked to withhold their anti-Parkinson’s disease medication, however, the collection of each pulse geometry condition as well as amplitude was randomized relative to medication administration across participants. Further research would be needed to appreciate whether the effects of manipulating pulse geometry are impacted by the presence or absence of dopamine medication. However, prior preclinical work has indicated that medication does not impact the short-latency responses (42). These data may differ from results reported from long-term, chronically implanted patients due to changes in lead positioning and disease fluctuations with time (65). Participants may also be impacted by the micro-lesioning effect and show a reduced level of motor symptoms as a result. A lack of behavioral data to demonstrate the clinical efficacy of each pulse geometry restricts these findings to the upper bounds of the therapeutic window as only side effects can be appreciated through the data reported in this study. A total of eight participants were included in this study, which may limit the ability to provide a detailed analysis of differences across individuals and variability due to lead position. Due to the limited time available for testing, only the contacts selected during the monopolar review could be assessed along with a single pulse width and stimulation frequency. Other contacts, pulse widths, or frequencies of stimulation may provide different response curves given different positions within the region of the STN and ability to activate the basal-ganglia thalamocortical circuit and other adjacent pathways. Given the pro-longed artifact during the CPF condition the short-latency peaks could not be quantified, which most closely approximate those found in clinical pulse generators.

## Data availability statement

The raw data supporting the conclusions of this article will be made available by the authors, without undue reservation.

## Ethics statement

The studies involving humans were approved by Cleveland Clinic Institutional Review Board. The studies were conducted in accordance with the local legislation and institutional requirements. The participants provided their written informed consent to participate in this study.

## Author contributions

BC, AM, and KB: conceptualization. BC, LF, RR, SN, and AM: investigation. BC, LF, JT, OH, and DE: formal analysis. BC, LF, JT, and OH: visualization. AM and KB: funding acquisition. DE, AM, and KB: supervision. BC: writing—original draft. BC, LF, JT, OH, SN, RR, DE, AM, and KB: writing—review and editing. All authors contributed to the article and approved the submitted version.

## Funding

This work was supported by the Farmer Family Foundation.

## Conflict of interest

AM was a consultant and had intellectual property licensed to Enspire DBS, had distribution rights in Ceraxis, and was a consultant to Abbott. KB was a consultant for Enspire DBS. AM and KB had intellectual property and distribution rights in Cardionomics. SN consulted for Abbott and was a speaker for Medtronic.

The remaining authors declare that the research was conducted in the absence of any commercial or financial relationships that could be construed as a potential conflict of interest.

## Publisher’s note

All claims expressed in this article are solely those of the authors and do not necessarily represent those of their affiliated organizations, or those of the publisher, the editors and the reviewers. Any product that may be evaluated in this article, or claim that may be made by its manufacturer, is not guaranteed or endorsed by the publisher.
