# Supplementary figures and images for: Myogenic and cortical evoked potentials vary as a function of stimulus pulse geometry delivered in the subthalamic nucleus of Parkinson’s disease patients
Source: Front Neurol. 2023 Aug 24;14:1216916. doi: 10.3389/fneur.2023.1216916 (PMC10484227; doi:10.3389/fneur.2023.1216916)

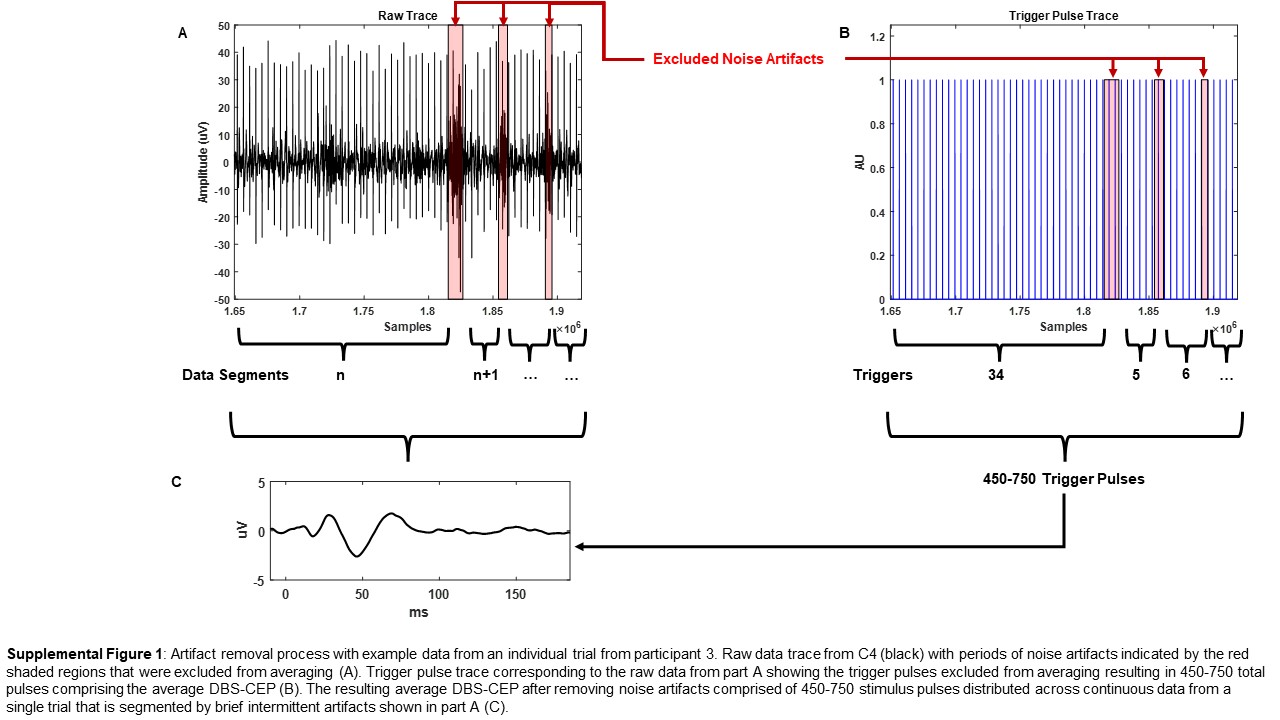

Supplement: Supplementary file 1 [file Image_1.JPEG]
